# Fibroblast growth factor 21 potentially inhibits microRNA-33 expression to affect macrophage actions

**DOI:** 10.1186/s12944-016-0381-6

**Published:** 2016-12-01

**Authors:** Yuan Guo, Fei Luo, Yuhong Yi, Danyan Xu

**Affiliations:** Department of Cardiovascular Medicine, The Second Xiangya Hospital, Central South University, Changsha, 410011 Hunan China

**Keywords:** Fibroblast growth factor 21, MicroRNA-33, Atherosclerosis

## Abstract

Atherosclerosis is a chronic inflammatory disease with complex pathological processes. MicroRNA-33 (miR-33), a novel non-coding RNA that coexpresses with sterol regulatory element-binding proteins (SREBPs), affects macrophage actions to prevent atherosclerosis. Fibroblast growth factor 21 (FGF21) is an important regulator of lipid metabolism, especially for macrophage-related cholesterol export, but the mechanism is not fully studied. Interestingly, FGF21 has been evidenced to prevent atherosclerosis via inhibiting SREBP-2 expression. Therefore, we speculate that FGF21 may be a potential regulator for miR-33 with an aim of insight into novel anti-atherosclerotic mechanisms and research fields.

## Introduction

Atherosclerosis is a chronic inflammatory disorder characterized by the deposition of excess lipids in the arterial intima. Reverse cholesterol transport (RCT) could counteract the pathogenic events by promoting cholesterol efflux to high-density lipoprotein (HDL) from the artery wall, which involves in a series of factors including macrophage or non-macrophage related cholesterol efflux, cell membrane bound transporters, plasma lipid acceptors, plasma proteins and enzymes, and hepatic cellular receptors [1]. Macrophages exert important roles in cholesterol efflux and preventing atherosclerosis. Cholesterol efflux from atherosclerotic plaque is a featured function of macrophage, which is determined by two critical transporters on the membrane. One is ATP-binding cassette transporter A1 (ABCA1), mainly interacting with cholesterol-deficient and phospholipid-depleted apolipoproteinA-I (apoA-I) complexes. The other is ATP-binding cassette transporter G1 (ABCG1) that mediates macrophage cholesterol efflux through interacting with spherical, cholesterol-containing alpha HDL particles [1]. Hence, increasing the two transporters on macrophages is critical to promoting RCT process and reversing atheroma.

Inflammation response is of central importance for the initiation and progression of atherosclerosis. Macrophage counts in symptomatic carotid plaques are significantly increased and considered to discriminate between symptomatic and asymptomatic patients [2]. Moreover, macrophage phenotypes have been identified as important factors in atherosclerotic conditions [3]. Accordingly, macrophages in atherosclerotic plaque are divided as pro- and anti-inflammatory macrophages, with polarization phenotypes M1 and M2 macrophages respectively. Both the two kinds of macrophages are hallmarks of atherosclerotic lesions. M1 macrophages are the predominant phenotype in rupture-prone shoulder regions while M2 macrophages present more stable of atherosclerotic plaque and prevent foam cell formation [3, 4]. It has been found that macrophages could alter their phenotypes and functions in response to certain stimulators or cytokines [5]. Thus, regulators to increase anti-inflammatory macrophages and decrease pro-inflammatory macrophages within atherosclerotic lesions are promising approaches to prevent atherosclerosis and plaque rupture.

MicroRNAs (miRNAs) are small, non-coding RNAs that regulate gene expression and control a wide range of biological functions by base pair with specific mRNAs. Recent reports have identified specific miRNAs as major regulators of lipid homeostasis and anti-atherosclerosis; and the best-characteristic one is miRNA-33 (miR-33) [6]. Although several recent findings reported that suppression of miR-33 is controversial in lipid metabolism and anti-atherosclerosis [7–9], more studies evidenced that inhibiting miR-33 could improve lipid profile and have atheroprotective properties. Rayner et al. [10] demonstrated that inhibition of miR-33 stabilized atherosclerotic plaques, and anti-miR-33–treated mice showed 35% reduction in plaque size and lipid content after 4 weeks, which was consistent with the levels of increased circulating HDL and enhanced RCT to plasma, liver, and feces. Rotllan et al. [11] further reported that long-term anti–miR-33 therapy for 12 weeks also significantly reduced the progression of atherosclerosis in low-density lipoprotein (LDL) receptor-deficient (Ldlr-/-) mice model.

Well-documented evidences of miR-33-induced anti-atherosclerotic mechanisms are focused on regulating macrophage actions. MiR-33 was reported to inhibit the expression of ABCA1 and ABCG1 in macrophages, thereby attenuating cholesterol efflux to apoA-I and nascent HDL [12]. While inhibiting miR-33 increases macrophage related cholesterol efflux by upregulating the expression of ABCA1 and ABCG1. Rayner et al. [10] found that anti-miR33–treated 4 weeks had a 66% increase in lesional macrophage ABCA1 expression compared with these control groups in mice. Besides, miR-33 was also identified to regulate macrophage polarization and thereby stabilizing atherosclerotic plaque to reduce cardiovascular events. Ouimet et al. [13] reported that in *Ldlr-/-* mice model, inhibition of miR-33 could delay progression of atherosclerosis by inhibiting monocyte recruitment and changing macrophage-induced inflammation in atherosclerotic plaques, which was independent of the effect of ABCA1 induced cholesterol export. Thus, inhibiting miR-33 could promote macrophage conversion from pro-inflammatory M1 to anti-inflammatory M2 phenotype to prevent atherosclerosis and stabilize plaque.

Fibroblast growth factor 21 (FGF21), a member of the fibroblast growth factor (FGF) family, has been described as an important regulator of glucose and lipid metabolism [14–16]. Recently, serum FGF21 levels was found positively related to coronary heart disease (CAD) and atherosclerosis. Chow et al. [17] in a cohort consisted of 670 subjects found that serum FGF21 levels positively correlated with carotid atherosclerosis in humans on multiple stepwise regression analysis. In another cohort study with 253 subjects, Shen et al. [18] further observed that subjects with CAD showed significantly higher serum FGF21, which was also positively correlated with total cholesterol (*P* < 0.05) and triglyceride (*P* < 0.01). Thus, serum FGF21 elevation under atherosclerosis conditions may be a regulatory compensation mechanism and indicate a promising therapeutic target.

Mechanically, FGF21 was also received wide attention in the effect of macrophage-derived cholesterol efflux. Shang et al. [19] found that in vitro treatment with FGF21 at 50 and 100 ng/mL significantly enhanced cholesterol efflux compared with the control group (*P* < 0.05); and this process was related to upregulating the expression of ABCA1 and ABCG1 in THP-1 macrophage-derived foam cells. Similarly, Lin et al. [20] reported that FGF21 promoted cholesterol efflux and ABCA1 expression in THP1 macrophage-derived foam cells in a dose- and time-dependent manner. While the mechanisms of how FGF21 increases ABCA1 and ABCG1 expression have not been completely clarified.

## Hypothesis

Sterol regulatory element–binding proteins (SREBPs) are a family of membrane-bound transcription factors that mainly regulate lipid homeostasis [21]. SREBPs have two isoforms including SREBP-1 and SREBP-2. Generally, SREBP-1 is induced by insulin resistance or hyperinsulinemia that leads to fatty acid and triglycerides synthesis, while SREBP-2 increases endogenous cholesterol synthesis [21]. Intriguingly, the two members of miR-33, miR-33a and miR-33b, are respectively located within the intron 16 of *SREBP-2* and the intron 17 of *SREBP-1* genes [22]. And miR-33 was reported to maintain cellular lipid level and cholesterol export by co-expressing with *SREBPs* gene [23]. Hence, regulators decreasing SREBPs expression are also considered as inhibitors of miR-33.

More interestingly, recent findings indicated that FGF21 regulate cholesterol efflux by targeting *SREBP-2* gene. Lin et al. [24] investigated the function of FGF21 in atherosclerosis. And they found that FGF21 deficiency caused a markedly increasing mortality of *apoE-/-* mice and exacerbation of atherosclerosis followed by significantly worsened lipid profile and inflammatory cytokines. In mechanism, they found that SREBP-2 was FGF21 targeted gene to regulate cholesterol efflux. Therefore, we speculate that FGF21 could inhibit SREBPs expression as well as the expression of miR-33.

**Fig. 1:**
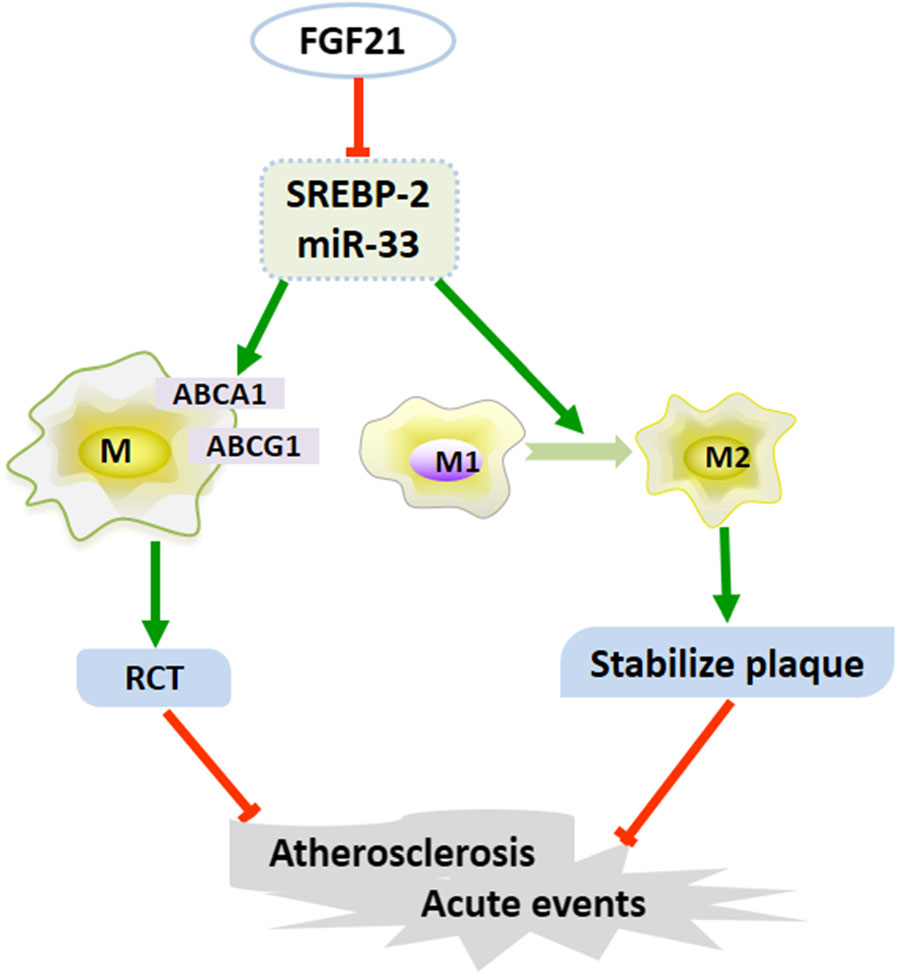
FGF21 potentially inhibits miR-33 expression to affect macrophage actions and prevent atherosclerosis. FGF21: Fibroblast growth factor 21, SREBP-2: Sterol regulatory element-binding proteins 2, ABCA1: ATP-binding cassette transporter A1, ABCG1: ATP-binding cassette transporter G1, RCT: Reverse cholesterol transport

Together, we hypothesis that FGF21 potentially inhibits miR-33 expression, and thereby enhancing macrophage related cholesterol efflux and increasing anti-inflammatory macrophages to prevent atherosclerosis (Fig. 1). This hypothesis aims to reveal a new potential mechanism of FGF21-induced anti-atherosclerosis by promoting macrophage actions. Besides, this hypothesis also try to provide new research directions for the conflicting findings of miR-33. As previously described, the function of miR-33-induced lipid-lowering and anti-atherosclerosis was in dispute due to several studies did not observed anti-atherosclerotic effects or even found elevated triglyceride levels. But Näär et al. [25] considered that this result might be partly related to miR-33b coexpressing with SREBP-1, which is a gene involving in insulin resistance and the feedback mechanism should responsible for the increased triglycerides level. Although it was reported FGF21 not only influenced SREBP-2 but also regulated SREBP-1 [26], FGF21 induced cholesterol efflux was mainly regulated SREBP-2 but not affected SREBP-1 [24]. Thus, studies to discuss the role of FGF21 in cholesterol export and macrophage behaviors may make more sense.

## Abbreviations

ABCA1: ATP-binding cassette transporter A1
ABCG1: ATP-binding cassette transporter G1
apoA-I: apolipoproteinA-I
CAD: Coronary heart disease
FGF21: Fibroblast growth factor 21
HDL: High-density lipoprotein
LDL: Low-density lipoprotein
RCT: Reverse cholesterol transport
SREBPs: Sterol regulatory element–binding proteins
